# Connolly, K. *Perceptual Learning: The Flexibility of the Senses*

**DOI:** 10.1177/0301006620943840

**Published:** 2020-07-20

**Authors:** Oliver Braddick, Janette Atkinson

**Reviewed by:** Oliver Braddick, *University of Oxford, Oxford, UK*; and Janette Atkinson, *University College London, London, UK*

Entering the topic “perceptual learning” into Web of Science returns over 100 publications for every year since 2008, so it is clearly a hot topic. And that probably doesn’t include most of the deluge of papers which use machine learning to distinguish stimulus categories—an endeavor which suggests the amazing potential of learning mechanisms and may provide models for human acquisition of perceptual abilities.

Kevin Connolly is a philosopher of mind, and he describes the aim of his book to “offer an empirically informed account of perceptual learning for philosophers.” The readership of *Perception* is not therefore his main target audience, but we can ask: how well does he present empirical perceptual science to philosophers? And, does his philosophical approach offer an analysis that is useful for empirical perceptual scientists?

Connolly has certainly done his homework on the empirical science—about 50% of the references cited are to studies in experimental psychology and neuroscience. However, in presenting these results, he does not necessarily convey the questions the scientists were asking. The first issue Connolly addresses is: “Does perceptual learning exist,” by which he means “Are the changes described by this term truly perceptual?” While he resoundingly refutes some philosophers who have denied that they are, he sets a boundary on “perception” of which we may be sceptical or impatient. For instance, he cites experiments by Law and Gold (2008) which found that the brain changes when macaques learned to improve their motion coherence thresholds were in decision processing structures rather than early visual areas and concludes that this disqualifies the learning in this case from being “perceptual.” The result is significant, but the implied dividing line may be less so—the more we know about the brain networks linking vision to decision, action, and recognition, the more we recognize the essential continuity of these processes, especially given the ubiquitous feedback loops that infuse information from “higher” areas into early visual processing. So Connolly cites several lines of evidence that learning modifies functional magnetic resonance imaging and electroencephalography responses in early visual cortex, but doesn’t ask from whence these effects find their way into early visual cortex. Questions about how the mechanisms of perceptual learning interface with the detailed processes of vision do not figure strongly in the issues he considers.

Certainly, perceptual scientists don’t necessarily draw the dividing line in the same place. It is notable that Connolly doesn’t cite the work of Barbara Dosher and her colleagues (Dosher & Lu, 2017) who conclude that “reweighting evidence from one level to another of representation (of which their major model is reweighting the inputs to a decision process) or within levels is a major and perhaps the major form of perceptual learning.” However, Connolly’s arguments, developed at length in several chapters, do feature “attentional weighting” as a major component of perceptual learning, so there is certainly scope for bridging the conceptual gap between the disciplines.

Attentional weighting is only one of three types of process which Connolly proposes as species of perceptual learning. The other two are “unitization”—the combination of elements (within or between modalities) into an integrated representation, and “differentiation”—the process of establishing distinct representations of two stimulus categories that are initially indistinguishable. From a mechanistic point of view, it is not clear how differentiation differs from attentional reweighting—the learning process comes to select and amplify the internal signals which are different between the categories (and perhaps downregulate those that they have in common). But Connolly is more concerned with phenomenology than mechanism and spends a lot of time on issues such as whether we come to *hear* the difference between homophone words such as “bank” (the financial institution) and “bank” (the edge of the river)—an instance of the “Phenomenal Contrast Argument” that is a target for his repeated refutation.

A surprising blind spot in Connolly’s account is early perceptual development. He accepts an assertion by Kellman and Garrigan that infants’ development of visual abilities arises primarily from “innate or maturational” mechanisms and doesn’t count as perceptual learning. This does not set easily with his awareness of the plasticity of the visual system in an initial critical period, nor with (to take an example) the development of face perception following the neonate’s sensitivity to a crude configuration, linked to intense exposure to human faces in the first months. Perhaps the problem is that babies don’t produce a commentary on their subjective visual experience, and develop vision in a way that is inextricably entangled with their acquisition of physical and social concepts and of visuo-motor skills.

Connolly’s discussion considers at various points the question of cognitive penetrability—how far our perceptual processes can be accessed, and manipulated, by consciously willed actions. It is an important issue, at least if we want to understand the relation between consciousness and the organization of the brain’s operation, and concerns psychologists and neuroscientists as well as philosophers. For Connolly, it connects with a provocative and (to these reviewers) implausible proposal. He suggests that the function of perceptual learning is to offload demands from slow, deliberate, and demanding cognitive processes to fast, automatic and highly parallel perceptual processes. This is a well-established idea in skill learning (remember needing to think about the gear lever when you were learning to drive). However, in the context of perceptual learning, it suggests that before learning has occurred, the judgments involved can be laboriously made through cognitive effort. One of Connolly’s favored examples is learning to perceive the difference between different wines. Are we to believe that the novice can distinguish Chateau Lafite from Chateau Margaux, if only she thinks about it long and hard enough? Or that Law and Gold’s monkeys could have, but didn’t, use cognitive processes to get their rewards for distinguishing 25% motion coherence early in their testing? Isn’t it good enough simply to suppose that we possess a potential learning process that enables us to see, hear, or taste differences that we simply couldn’t see, hear or taste before?

As perceptual scientists, we must welcome that philosophers of mind are bringing relevant experimental evidence to bear on the questions they debate. And eavesdropping on their debates can and should provoke experimentalists to think harder about the meaning of their results. But a book such as this, in the end, reminds us that most of the time, we are asking different questions.
